# Mirolysin structures open a window on gum disease

**DOI:** 10.1107/S2052252519016968

**Published:** 2020-01-01

**Authors:** Evette S. Radisky

**Affiliations:** a Department of Cancer Biology, Mayo Clinic, Jacksonville, Florida 32224, USA

**Keywords:** periodo­ntitis, peptidase, protease, crystal structure, zymogen activation, substrate specificity

## Abstract

Guevara, Rodriguez-Banqueri *et al.* [(2020), *IUCrJ*, **7**, 18–29] determine crystal structures of mirolysin, a metalloprotease that helps oral pathogen *Tannerella forsythia* evade the human immune response. The structures provide insight into the regulation and specificity of mirolysin, and hint at how it might be inhibited for therapeutic effect.

Periodo­ntitis, commonly known as gum disease, is a chronic inflammatory disease characterized by dysregulation of the oral microbiota in the subgingival space below the gum line (Hajishengallis, 2015 ▸). Healthy gums are compromised by a proliferation of pathogenic anaerobic bacteria, most commonly the triad of *Porphyromonas gingivalis*, *Treponema denticola* and *Tannerella forsythia*. These pathogens release ‘virulence factors’, molecules that aid their colonization and survival in the subgingival niche and their evasion of the host immune response. Synergistic interactions of the pathogenic microbes with normal oral microbiota lead to a sustained host inflammatory response, degrading periodo­ntal tissues, eroding bone, and ultimately leading to tooth loss. Severe periodo­ntitis affects around 10% of the human population, and health implications are not limited to oral health; the chronic inflammatory conditions precipitated by the disease can extend to systemic manifestations, contributing to risk of cardiovascular and respiratory diseases, diabetes, rheumatoid arthritis, and cancer (Hajishengallis, 2015 ▸; Peters *et al.*, 2017 ▸).

Proteases are amongst the most abundant virulence factors produced by periodo­ntal pathogens. These enzymes degrade host proteins to feed bacterial growth, and also serve as key mediators of immune-subversive mechanisms, for example by degrading or inactivating endogenous antimicrobial peptides and components of the host complement system (Hajishengallis, 2015 ▸). One of the proteases implicated in these activities is mirolysin, a metalloprotease secreted by *T. forsythia*. Mirolysin cleaves multiple components of the complement pathway to protect the pathogen against complement-mediated killing, and degrades the antimicrobial peptide LL-37, a human host defense peptide secreted by epithelial and immune cells at sites of infection (Jusko *et al.*, 2015 ▸; Koneru *et al.*, 2017 ▸). As a recently discovered mediator of *T. forsythia* survival and immune evasion, mirolysin offers a potential therapeutic target, yet until now the structural basis for its latency, activation and substrate specificity have not been defined.

In this issue of **IUCrJ**, Guevara, Rodriguez-Banqueri *et al.* report high-resolution crystal structures of promirolysin, defining the mechanism of latency of the precursor protein (Guevara *et al.*, 2020 ▸). Like nearly all proteases, mirolysin is first produced as a zymogen, maintained in an inactive state by the presence of an N-terminal extension known as a pro-peptide, pro-segment or pro-domain (Fig. 1 ▸). Upon proteolytic cleavage of the pro-segment, the active site of a protease becomes exposed and accessible to substrates. While unified by this common theme, different clans and families of metallo­proteases show myriad variations in the details of how latency is maintained and activation subsequently achieved. These variations have been carefully cataloged in over 60 crystal structures of metalloprotease zymogens, including many from the Gomis-Rüth research group (Arolas *et al.*, 2018 ▸). Here, the group finds that the mechanism of latency of promirolysin closely resembles the cysteine switch mechanism of matrix metalloproteinases (MMPs) and a number of other zinc metallopeptidases (Morgunova *et al.*, 1999 ▸; Arolas *et al.*, 2018 ▸). A stretch of the pro-segment occupies the enzyme active site cleft in an orientation opposite to that of a productively bound substrate, positioning a conserved cysteine residue to bind the catalytic zinc. The cysteine side chain displaces the normally bound water molecule that plays the essential hydrolytic role in proteolysis, thus rendering the zymogen inactive.

While this basic cysteine switch mechanism of latency is well precedented, the promirolysin structure reveals a surprisingly extensive interface between the pro-segment and the catalytic domain. The zymogen structure reveals a buried surface area of 2302 Å^2^ between these structural elements, greater than the average buried surface area for protein–protein complexes, which is remarkable given the small size of the 34-residue pro-segment. The pro-segment and catalytic domain interact through a balance of favorable hydro­phobic interactions complemented by an array of hydrogen bonds and salt bridges, the same forces that shape affinity and selectivity of intermolecular protein–protein interactions (Xu *et al.*, 1997 ▸). The extensive nature of the broad interface between pro-segment and catalytic domain suggests the possibility that the pro-segment may be capable of inhibiting the enzyme *in trans*, and serving as a template for engineering protein-based mirolysin inhibitors of therapeutic utility. Similar approaches have shown promise for engineering therapeutic inhibitors of ADAM family metallopeptidases, based upon their much larger pro-domains (Moss *et al.*, 2007 ▸; Wong *et al.*, 2016 ▸).

Guevara, Rodriguez-Banqueri *et al.* further report a high-resolution structure of active mirolysin bound to a peptide product, providing insight into determinants of substrate specificity of the protease (Guevara *et al.*, 2020 ▸). A preference of mirolysin for cleavage on the N-terminal side of basic residues (Koneru *et al.*, 2017 ▸) is explained by the presence of an aspartate residue positioned to form a salt bridge with the substrate. This aspartate residue is conserved among the pappalysin family of metalloproteases to which mirolysin belongs, suggesting that similar specificity may be typical of the entire family, and that the mirolysin structure can offer a model to understand substrate recognition for the larger family. Additionally, this serendipitously trapped product complex may provide yet another starting point from which active site-targeted mirolysin inhibitors might be developed. A similar serendipitous discovery of a product fragment captured in a crystal structure of MMP-13 recently led to the design of a potent peptidomimetic inhibitor of this metalloprotease (Gall *et al.*, 2019 ▸). Together the new structures of mirolysin reported here offer a window from which to understand the function of this protease in gum disease, with a glimpse of how one might inhibit this intriguing target.

## Figures and Tables

**Figure 1:**
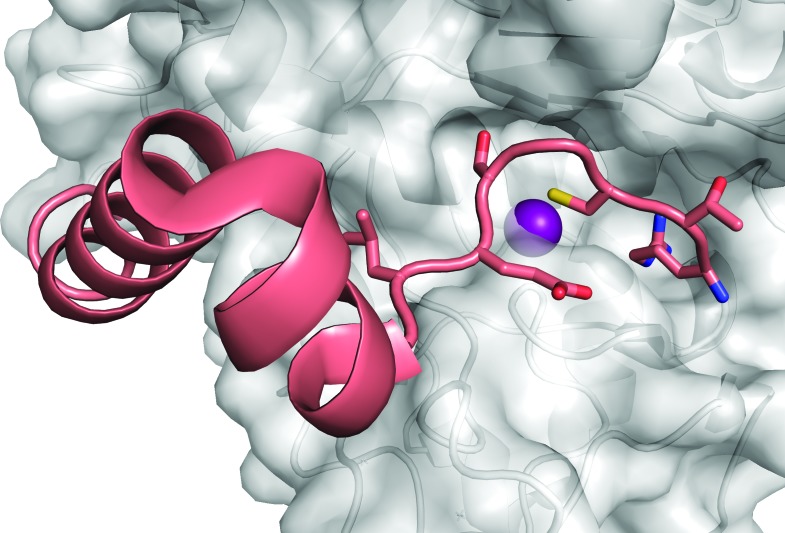
Structure of promirolysin reveals how the pro-segment inhibits enzyme activity prior to proteolytic activation. The N-terminal residues of the pro-segment (salmon) occupy the active site cleft of the catalytic domain (gray), with a key cysteine residue coordinating the catalytic zinc ion (purple). This figure was generated using *Pymol* from the coordinates of PDB structure 6r7v, reported in this issue by Guevara, Rodriguez-Banqueri *et al.*
